# Epidemiology of Non-Contact Muscle Injuries in the Italian Male Elite Under-19 Football (Soccer) Championship

**DOI:** 10.1186/s40798-024-00738-0

**Published:** 2024-06-21

**Authors:** Massimo Magistrali, Luca Stefanini, Michele Abate, Giulio Biancalana, Andrea Stegagno, Paolo Cugia, Piero Candoli, Giuseppe Anania, Pier Luigi Lucchese, Diego Gaddi, Piero Volpi, Francesco Mariani, Lorenzo Boldrini, Nicola Filippi, Annunziata Cerrone, Cristiano Sirtori, Paolo Battaglino, Guido Bravin, Emilio Del Fabro, Mattia Berti, Eugenio Vecchini, Marco A. Minetto

**Affiliations:** 1 J|medical, Torino, Italy; 2 Juventus FC, Torino, Italy; 3 IRCCS San Raffaele Hospital, Milano, Italy; 4 Bologna, Bologna, 1909 Italy; 5 Cagliari Calcio, Cagliari, Italy; 6 FC Cesena, Cesena, Italy; 7 FC Empoli, Empoli, Italy; 8 Frosinone Calcio, Frosinone, Italy; 9 Istituto Clinico Humanitas IRCCS Rozzano, Milano, Italy; 10 FC Internazionale Milano SpA, Milano, Italy; 11 Department of Orthopedics and Traumatology, Policlinico San Pietro, Ponte San Pietro, Bergamo, Italy; 12 Transalpine Center of Pediatric Sports Medicine and Surgery, University of Milano-Bicocca, Monza, Monza-Brianza, Italy; 13 MilanLab Research Department, AC Milan, Milano, Italy; 14 AC Milan, Milano, Italy; 15 SSC Napoli, Napoli, Italy; 16 UC Sampdoria, Genova, Italy; 17 FC Torino, Torino, Italy; 18 SOC Ortopedia e Traumatologia ASUFC, Udine, Italy; 19 Udinese Calcio, Udine, Italy; 20 Department of Knee Surgery, Policlinico Abano Terme, Abano Terme, Italy; 21 Hellas Verona Calcio, Verona, Italy; 22 Clinica Ortopedica, University of Verona, Verona, Italy; 23 Division of Physical Medicine and Rehabilitation, Department of Surgical Sciences, University of Turin, Torino, Italy

**Keywords:** Hamstrings, Adductors, Iliopsoas, Incidence, Burden, Distribution

## Abstract

**Background:**

While extensive research exists on muscle injuries among adult football players, a notable gap persists in studies concerning younger footballers. The aim of the current study is to provide epidemiological data on the characteristics of time-loss muscle injuries in young football players participating in the Italian Under-19 male elite Championship (“Primavera 1”).

**Results:**

Conducted as a multicentre, prospective, observational cohort study, this research gathered injury data from the 2022-23 season across 14 of the 18 Clubs in the first Italian Under-19 championship. The cohort comprised 391 players with a mean age (± standard deviation) of 18.0 ± 0.4 years. A total of 479 injuries were reported, resulting in 14,231 days of activity lost. Of these, muscle injuries were 209 (44%), accounting for 4,519 (32%) days lost. Overall muscle injuries incidence was 1.82/1000 hours, with a mean injury burden of 39.4 days lost/1000 hours. Almost all muscle injuries (206 out of 209: 98.5%) occurred in hamstrings, quadriceps, adductors, calf and iliopsoas. Hamstrings injuries were the most burdensome (18.8 days lost/1000 hours) accounting for nearly half of all days lost due to muscle injuries. Incidence and burden of adductors injuries (0.25 injuries and 4.1 days lost/1000 hours, respectively) were found to be comparable to calf injuries (0.24 injuries and 4.7 days lost/1000 hours, respectively). Iliopsoas injuries accounted for a noteworthy portion of the total, with an injury incidence of 0.16/1000 hours and a burden of 3.3 days lost/1000 hours. Injuries with myo-tendinous or myo-aponeurotic involvement demonstrated delayed return-to-football compared to those without such involvement (35.6 vs. 18.5 days, *p* < 0.0001).

**Conclusions:**

The study highlighted a peculiar distribution of non-contact muscle injuries among elite young football players. While hamstring injuries were confirmed as the most burdensome, incidence and burden of adductors and calf injuries were found to be similar. A significant incidence and burden of iliopsoas injuries were observed. These findings suggest potential implementations for targeted injury prevention strategies in the Italian male elite Under-19 football Championship.

**Supplementary Information:**

The online version contains supplementary material available at 10.1186/s40798-024-00738-0.

## Key points

• In the Italian male elite Under-19 football Championship, each Club can expect around 15 non-contact muscle injuries during the season (for a team of 25 players) distributed as follows: 6 hamstrings, 3 quadriceps, 2 adductors, 2 calf and 1 iliopsoas muscle injuries.

• Hamstrings injury incidence and burden (both in training sessions and during matches), as well as the proportion of reinjuries, were found to be the highest among muscle sites.

• Injuries to adductors and calf muscles exhibited comparable values of incidence and burden.

• Iliopsoas emerged as a noteworthy site of injury in the current cohort, particularly during training sessions.

• Aponeurosis or tendon damage was associated with longer return to football timing, particularly in hamstrings and adductors injuries.

**Supplementary Information:**

The online version contains supplementary material available at 10.1186/s40798-024-00738-0.

## Background

Football (soccer) is recognized globally as the most widely practiced sport, particularly among the youth [1]. Young football players face a high incidence of injuries [2] that could significantly impact their health and future careers [3, 4]. Understanding the incidence, burden, and characteristics of injuries within this age group is therefore crucial for developing effective prevention strategies [5, 6].

Numerous studies have examined the epidemiology of injuries in youth football, yet challenges persist in accurately characterizing these injuries. Firstly, the type and burden of injuries change with age during adolescence [7, 8], gradually aligning more with those observed in adults [9–11]. This developmental progression complicates data interpretation from studies that include multi-year age groupings [6, 12].Therefore, it may be advisable to conduct studies on narrower age groups. Secondly, data related to players’ exposure to training and match loads are paramount to calculate injury incidence and burden [13, 14]. Absolute values do not account for disparities in exposure between teams, resulting from different National and International tournaments in addition to the League. Other variables such as the attitudes of the coaching staff and geographic location also come into play, limiting the generalization of results from short-time studies and those focused on single Clubs [15, 16]. A multicentre study design is thus adviseable for mitigating these limitations. Previous studies involving multiple Clubs in the same league, however, rely on indirect sources such as the media [17, 18], with questionable reliability regarding injuries of moderate and mild prognosis [19]. Ultimately, investigations concerning this demographic lack standardized injury collection criteria and definitions. For these reasons, the available data on youth football injuries are better suited for providing a general overview of the magnitude of the issue rather than precisely characterizing it [2]. For instance, a number of studies provides data concerning the incidence of overall injuries in youth players [4, 6, 7, 20], yet to the best of our knowledge data on site-specific muscle injuries distribution are not reported. Non-contact muscle injuries, however, are the prevalent form of preventable injuries and require dedicated prevention programs [21].

This study seeks to provide useful clinical information and to bridge this gap in the literature by detailing characteristics, distribution, and impact of non-contact muscle injuries in players participating in the Italian elite Under-19 male youth championship, known as “Primavera 1”.

## Methods

### Study design and participants

**Fig. 1:**
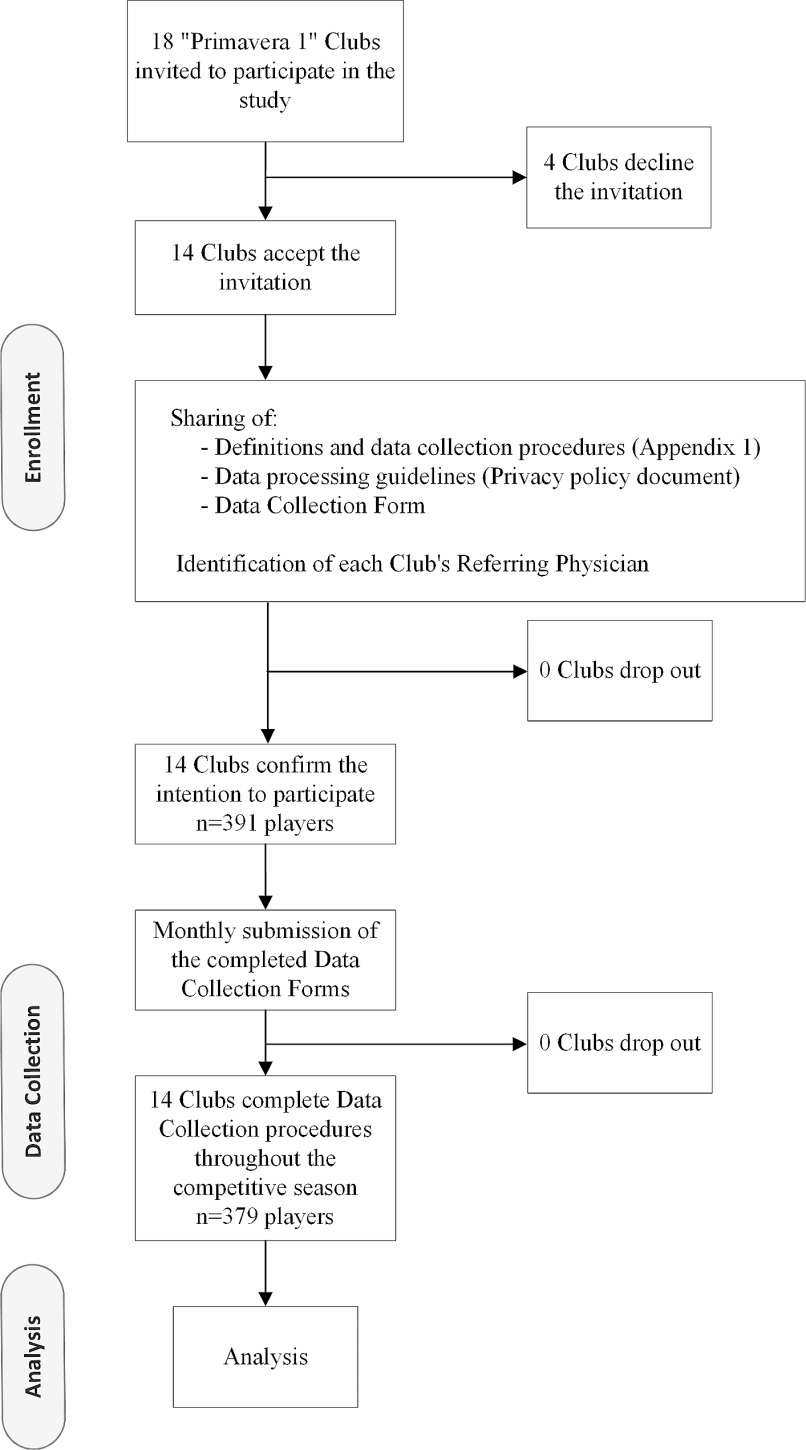
Study flow chart

The present investigation was structured as a multicentre, prospective, observational cohort study. All 18 Clubs competing in the first series of the Italian Under-19 football Championship during the 2022-23 season were invited to participate. Of the 14 Clubs that joined the initiative, all shared the requested data as described below. The remaining four Clubs opted not to participate in the study (Fig. 1). The study cohort consisted of 391 male players with a mean (± standard deviation) number of players per team of 27.9 ± 1.9. Their anthropometric data (collected in the pre-season evaluation) were: age 18.0 ± 0.4 years (range 16–24); weight 74.9 ± 1.7 kg; height 181.5 ± 2.9 cm. After the January transfer window, the cohort was reduced to a total of 379 players. Data concerning players who dropped out of the Championship were considered up until the moment of their exclusion.

Written informed consent for study participation was obtained from all participants. The study conformed to the guidelines of the Declaration of Helsinki and was approved by the Ethics Committee of the University of Turin (protocol n. 0574321).

### Study procedures

The study procedures followed the previously published Clinical Practice Guidelines and Consensus Statements on definitions and data collection procedures in studies of football injuries [13, 14, 20]. For each club, a referring physician (RP) was identified for correspondence and injury records clarifications if needed. Prior the start of the study, each RP received a pre-defined table to be completed and forwarded electronically to the study coordinator (MM) on monthly basis. Each RP held a medical degree and possessed experience in youth football (ranging from a minimum of 2 to a maximum of approximately 30 years of medical activity). All RPs were provided with a study manual describing definitions and procedures to be used (Appendix 1). A list of the definitions adopted in the current study is reported in Table 1.

**Table 1:** Specific details regarding the terminology adopted in the current study

Term	Description
Injury	Any tissue damage or physical integrity impairment reported by the player due to training or a football match, which would compromise his participation in team sports activities for at least the following two days (see Appendix 1)
Match exposure	Organised scheduled match played between opposing teams (not including internal training matches within the same team/club) [14] in minutes
Training exposure	Physical activities performed by the players aimed at maintaining or improving their skills, physical condition and/or performance in football [14] in minutes
Rehabilitation sessions	Not included in training/match exposure [14]
Return to football	The date when the injured player returns to full unrestricted team training without modifications in duration and/or activities [14]
Days lost of activity	Number of days that passed between the date of the injury occurrence and the date of return to football [54]
Reinjury	Subsequent injury in the same region and with the same diagnosis as the index injury, following full recovery and return to football [14]. Index injuries sustained before the start of a study were also considered [22]
Injury incidence	Number of injuries per 1000 player hours ((Σ injuries/Σ exposure hours)×1000) [14]
Injury burden	Number of days lost of activity per 1000 player hours ((Σ days lost/Σ exposure hours)×1000) [14]

Individual player characteristics (pre-season age, weight, height, limb dominance, position) and participation in matches and training sessions (minutes of exposures) as well as injuries were registered. The definition of injury used was “any tissue damage or physical integrity impairment reported by the player due to training or football match, which would compromise his participation in team sports activities for the following two days” (i.e., a modified version of time-loss injuries) [14]. Injuries that occurred during a match resulting in exclusion or substitution during the game were also included, regardless of the days lost due to injury. The investigated data for each injury comprised: date of injury, type of injury (categorized as Muscle injury, Traumatic injury, Overuse injury, Tendon injury, non-contact Sprain, other type of injury), occurrence during a training session or a match, date of return to unrestricted training with the team and days lost due to the injury. In case of injuries occurring at the end of the season, days lost were counted on a realistically estimated return to football date provided by the RP [22]. For muscle injuries, additional information on the involved muscle and tendon or aponeurosis involvement was recorded. Myotendinous/Myoaponeurotic (MT/MA) injuries were defined as injuries involving lesions of the myotendinous/myoaponeurotic junction of the muscle inclusive of the related connective tissue framework (tendon/aponeurosis) [23]. Injuries to muscle tissue resulting from direct contact, such as contusions or lacerations, were classified separately as Traumatic. Reinjuries were defined as subsequent injuries in the same region and with the same diagnosis as the index injury, following full recovery and return to football [14]. Index injuries sustained before the start of the study were also considered when identifying an injury as a reinjury [22]. The registration of a muscle injury was based on a clinical examination by the team medical staff, completed with further diagnostic assessment on a case-by-case basis at the discretion of the RP. Imaging studies performed for iliopsoas injuries were retrospectively requested to each RP due to the significant number of iliopsoas injuries reported. Exposure and injuries occurred by players with the National team were not collected.

### Analyses

Injury incidence was calculated as number of injuries per 1000 player hours of exposure [14]. The number of muscle injuries for each site in the lower limb was also reported as mean per team-season, considering a team composed of 25 players. Injuries were categorized under 4 degrees of severity based on the number of days’ absence [9]: (i) minimal (1–3 days), (ii) mild (4–7 days), (iii) moderate (8–28 days), (iv) severe (> 28 days). Injury burden was calculated as the mean number of days lost due to injury per 1000 h of athletic exposure [14]. Data were reported as means and standard deviation (SD) or 95% confidence interval (CI), as indicated [9]. The 95% CI for injury burden was computed via bootstrapping [24], with results based on 1000 bootstrap samples. The independent sample t-test was adopted for comparisons between different subgroups. A Spearman correlation rank was employed to assess the presence of an association between injury incidence and burden with the final ranking placement in the championship.

The threshold for statistical significance was set to *P* = 0.05. Statistical tests were performed with SPSS v. 20.0 (SPSS Inc., Chicago, IL, USA) software package.

## Results

A total of 479 injuries (mean ± SD: 34 ± 14 per team) resulting in 14,231 days lost (mean ± SD: 1016.5 ± 422.9 per team) were registered. The overall total exposure was 114,734 h (mean of 293.4 h per player), including 103,210 h of training and 11,524 h of match play. Each Club played a mean number of 46 matches per season (ranging from a minimum of 40 to a maximum of 57). The overall injury incidence was 4.17/1000 hours of exposure. Injury incidence was 6.3 times higher during match play compared with training (17.09 vs. 2.73/1000 hours, *P* < 0.001). No significant correlation was observed between the injury incidence or burden and final ranking placement in the Championship (*P* > 0.50 for both correlations). The distribution by type of injuries in absolute number and in days lost of activity is detailed in Table 2.

**Table 2:** Injuries distribution reported by type

	Number of injuries (%)	Days lost (%)
Overall	479	14,231
Muscle (non-contact)	209 (44)	4519 (32)
Traumatic	148 (31)	4886 (34)
Non-contact Sprains	48 (10)	2056 (14)
Overuse	40 (8)	1667 (12)
Tendon	31 (6)	994 (7)
Other type	3 (1)	109 (1)

### Muscle injuries

Muscle injuries constituted 44% (209 out of 479) of all reported injuries (mean ± SD: 14.9 ± 7 per team) resulting in a total of 4,519 days lost (mean ± SD: 322.8 ± 183 per team). From the 209 muscle injuries, 124 (59%) occurred during training and 85 (41%) during match play. The overall muscle injury incidence was 1.82/1000 hours (95% CI: 1.59–2.09). Muscle injury incidence was 6.1 times higher during match play compared with training (7.38 vs. 1.2/1000 hours, *P* < 0.001).

**Fig. 2:**
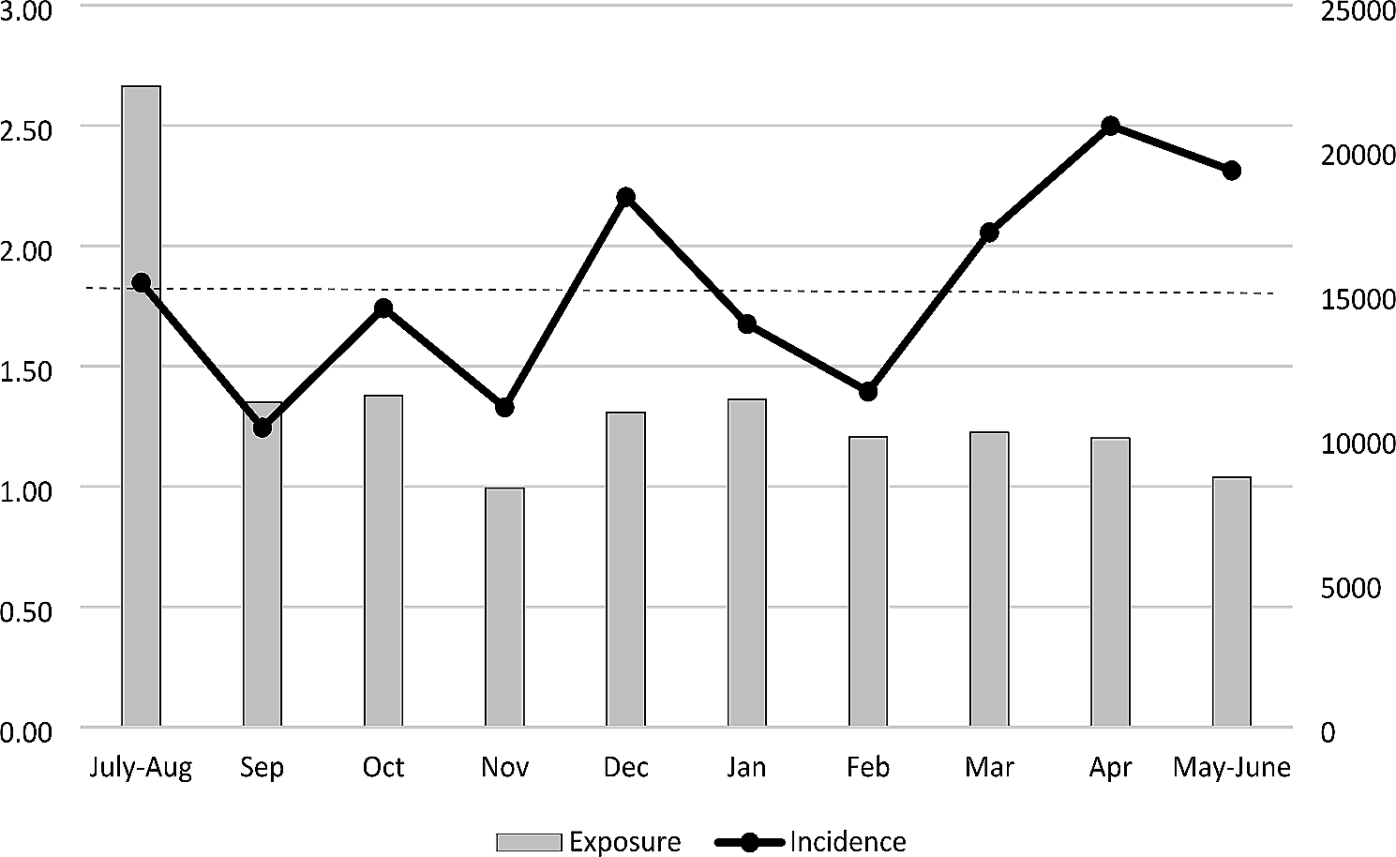
Monthly muscle injuries incidence in the 14 Clubs participating in the study Incidence (left vertical axis) = number of muscle injuries/1000 hours of exposure Exposure (right vertical axis) is reported in hours of football (training and match play) Dashed line = mean seasonal incidence of muscle injuries (1.82/1000 hours of exposure)

The monthly incidence of muscle injuries is reported in Fig. 2. The months with higher incidence were April and May-June (2.50 and 2.31 muscle injuries/1000 hours of exposure, respectively), while September had the lowest incidence (1.24 muscle injuries/1000 hours of exposure).

Almost all muscle injuries (206, equivalent to 98.5%) occurred in the following 5 sites: hamstrings (92), quadriceps (40), adductors (29), calf (27), and iliopsoas (18). The remaining 3 injuries affected abdominal muscles. All 18 iliopsoas injuries were clinically evaluated and assessed through further diagnostic investigations: 8 through ultrasound (44.4% of total iliopsoas injuries), 3 through MRI (16.7%), 7 through both (38.9%).

Incidence data, severity, injury burden and reinjuries are reported in Table 3. The majority of muscle injuries were of moderate severity, accounting for approximately half of the total (between a minimum of 41% for adductors and calf injuries and a maximum of 61% for iliopsoas injuries). Only two of the reported injuries involved a number of days lost between 91 and 180, with none exceeding 180. The site with the highest percentage of severe injuries was the hamstrings (29%), while the highest percentage of minimal and mild injuries was reported in the adductors (38%) and the calf muscles (37%). Each team lost a mean number of 323 days due to muscle injuries: 154 days (48%) for hamstring injuries, 68 days (21%) for quadriceps injuries, 39 days (12%) for calf injuries, 33 days (10%) for adductors injuries, 27 days (8%) for iliopsoas injuries and 2 days (0.6%) for other muscle injuries. Injury burden was therefore higher for hamstrings followed by quadriceps, calf, adductors and iliopsoas injuries. The adductors showed the lowest injury burden during training sessions and the highest match-to-training injury burden ratio (7.5). The muscle group with the highest proportion of reinjuries was the hamstrings, representing about 1 in 6 injuries in that area (18%).

**Table 3:** Muscle injuries characteristics MT/MA = Myotendinous/Myoaponeurothic involvement

	Total	Hamstrings	Quadriceps	Adductors	Calf	Iliopsoas	Others
Number (% muscular)	209	92 (44)	40 (19.1)	29 (13.9)	27 (12.9)	18 (8.6)	3 (1.4)
Incidence^1^, total	1.82 (1.59–2.09)	0.80 (0.65–0.98)	0.35 (0.26–0.47)	0.25 (0.18–0.36)	0.24 (0.16–0.34)	0.16 (0.10–0.25)	0.03 (0.01–0.08)
Incidence^1^, training	1.20 (1.01–1.43)	0.48 (0.37–0.64)	0.26 (0.18-0,38)	0.16 (0.10–0.26)	0.16 (0.10–0.26)	0.12 (0.07–0.20)	0.02 (0-0.08)
Incidence^1^, match play	7.38 (5.97–9.12)	3.64 (2.69–4.93)	1.13 (0.65–1.94)	1.04 (0.59–1.83)	0.87 (0.47–1.61)	0.52 (0.23–1.16)	0.09 (0.01–0.62)
Days lost, total	4516	2158	945	468	540	378	27
Days lost^2^	21.6 ± 19.5	23.5 ± 20.9	23.6 ± 19.4	16.1 ± 12.7	20 ± 18	21 ± 25.1	9 ± 7.8
Injury burden^3^	39.4 (28.9–56.9)	18.8 (12.2–28.5)	8.2 (4.7–14.8)	4.1 (2.7–6.4)	4.7 (2-7.5)	3.3 (1.1–7.5)	0.2 (0.1–0.7)
Injury burden^3^, training	27.5 (17.4–42.8)	12.4 (7.2–20.3)	6.5 (3.6–11.6)	2.5 (1-4.5)	3.1 (1.1–5.6)	2.8 (0.9–7.8)	0.2 (0.1–0.6)
Injury burden^3^, match play	145.6 (109.4-173.2)	75.9 (53.1-100.4)	24.1 (11.7–41.8)	18.6 (9.7–27.1)	19 (4.7–29.4)	7.6 (1.4–11.7)	0.3 (0.3–0.9)
Severity – number (%)							
Minimal	15 (7)	6 (7)	5 (13)	3 (10)	1 (4)	0	0
Mild	38 (18)	13 (14)	2 (5)	8 (28)	9 (33)	4 (22)	2 (67)
Moderate	105 (50)	46 (50)	24 (60)	12 (41)	11 (41)	11 (61)	1 (33)
Severe	51 (24)	27 (29)	9 (23)	6 (21)	6 (22)	3 (17)	0
Reinjuries – number (%)	24 (11)	17 (18)	4 (10)	2 (7)	1 (4)	0	0
MT/MA – number (%)	32 (15)	13 (18)	7 (21)	4 (22)	4 (24)	4 (29)	0
Days lost, MT/MA^2^	35.6 ± 25.2	40.5 ± 28.4	39.3 ± 31.1	28.8 ± 8.0	35.8 ± 24.2	20.3 ± 15.9	0
Days lost, non-MT/MA^2^	18.5 ± 16.5	24.5 ± 15.8	24.8 ± 13	14.1 ± 12.2	17.2 ± 15.8	21.2 ± 27.6	0

^1^Incidence is reported as number of non-contact muscle injuries/1000 player hours of exposure (95% CI)

^2^Data are reported as mean (± SD)

^3^Injury burden = number of days lost/1000 hours of exposure (95% CI) [24]

The mean number of expected muscle injuries for a team of 25 players can be estimated as follows: 15 injuries in total, 6 injuries for hamstrings, 3 injuries for quadriceps, 2 injuries for adductors, 2 injuries for calf, and 1 injury for iliopsoas muscle.

Table 3 also presents data regarding the MT/MA involvement in muscle injuries, observed in 32 cases (15.3%), with 174 cases (83.2%) exhibiting no involvement. The involvement status was unknown in 3 cases. The mean number of days lost for injuries with MT/MA involvement was significantly higher compared to injuries without MT/MA involvement (35.6 vs. 18.5 days, *P* < 0.0001). This difference remained statistically significant for the subgroup analyses relative to hamstrings (40.5 vs. 24.5 days, *P* = 0.02) and adductors injuries (28.8 vs. 14.1 days, *P* = 0.02).

## Discussion

This is the first prospective multicentre study investigating injury epidemiology in the Italian male elite Under-19 football Championship including fourteen Clubs participating in the League. The main results of the study can be summarized as follows: (i) the hamstrings were the most affected muscle group: injury incidence and burden (both in training and during matches), as well as the proportion of reinjuries, were found to be the highest among all muscle sites; (ii) injuries to adductors and calf muscles exhibited comparable values of incidence and burden; (iii) significant incidence and burden of injuries in the iliopsoas muscle, particularly during training sessions; (iv) the MT/MA involvement in muscle injuries was associated with a longer return-to-football timing.

The majority of the findings in the present study are aligned with those reported in professional football players: data related to overall injuries as well as to hamstrings, quadriceps, and calf muscles injuries are similar to those previously reported for adult players [2, 10, 11, 25].

The hamstrings emerge as the most affected muscle group, with absolute and percentage values as well as injury burden higher than in the past [10, 26] but lower than in the contemporary adult professional players [9]. The recurrence rate of hamstring injuries was found to be the highest among the different muscle groups, with values comparable to a recent study performed in a large group of adult professional players [9]. Reducing hamstring injuries thus represents a primary objective of prevention strategies. Despite being a long-recognized issue, epidemiological data in football demonstrate the challenges in achieving favourable outcomes in this muscle group [9], partly due to the increasing number of seasonal commitments [27]. Optimal return-to-football management requires multidisciplinary collaboration between coaching and medical staff with continuous and personalized assessments [28]. In this regard, a recent study highlighted how certain parameters may help identify players at higher risk of recurrence [29], allowing for focused prevention efforts. Implementing these processes is even more crucial in young soccer players, considering the season burden and the potential career-shortening effect of such injuries [30]. Quadriceps injuries are confirmed among the top three most frequent sites of muscle injuries, a typical feature of sports involving kicking and sprinting activities [31, 32]. Incidence and burden resulted similar to adults professional players [10]. The quadriceps is the most significant hip flexor after the iliopsoas. However, data indicating a possible compensatory decline in quadriceps injuries concomitant to the increase in iliopsoas injuries were not observed in the present study. These findings may support the hypothesis that the reported injury distribution is not the result of a different balance between the rectus femoris and iliopsoas in hip flexion in young footballers.

The adductor injury incidence and burden found in this study is much less, particularly in training, that what has been previously reported in professional football players. Usually, they are the second most important muscle group to be injured after the hamstrings, with an incidence of 0.6 to 0.8 per 1000 h and a burden of 8 days per 1000 h [10, 33]. Describing the possible reasons behind these results in a unique manner is not straightforward. In two prospective epidemiological investigations on football players [33, 34], adductors injuries resulted the most frequent cause of acute groin pain followed by iliopsoas injuries. These two muscle groups are not only anatomically but also functionally related. Adductor longus is the predominant site of acute muscle injuries among the adductors and assists in hip flexion, with the primary driver being the iliopsoas. Interestingly, the concomitant increase in the incidence of iliopsoas injuries and the decrease in adductor injuries in this study compared to the literature may suggest a potentially different functional balance between these two muscle groups in the current study. Age may thus be a primary causal factor behind this peculiar distribution. Therefore, it may be advisable to implement prevention strategies focused also on trunk control and hip flexors in younger athletes.

Incidence and burden of calf injuries in the current study are consistent with those reported in adult soccer players [10]. However, player’s older age has been reported as a risk factor for calf injuries [35]. In a large study, Ekstrand et al. reported a match incidence of 0.32/1000 hours of football in the 16–21 years age group [10]. In the present study, this figure appears higher (0.87/1000 hours of match play) with values similar to those reported in the 22–30 age group of the aforementioned study [10]. Data related to incidence and burden overall and during training sessions of calf injuries are not reported for each age group in the previous study, limiting the possibility of comparisons.

Accurate data regarding acute iliopsoas injuries are difficult to find in the literature. They are often accounted with hip issues [36, 37] or included in umbrella terms such as ‘hip flexors’ [36, 38] and ‘groin pain’ [33, 39]. Data reported in this study specifically refer to acute muscle injuries, separately reporting differential diagnoses such as iliopsoas-related groin pain, tendinopathy, bursitis or snapping. Verifying iliopsoas muscle injuries through imaging in all cases enhances the reliability of this finding, considering the intricate diagnostic process in the groin region. Iliopsoas muscle injuries appear to be almost exclusive to young athletes, particularly those involved in football and sprinting movements [38], while they seem exceptional in other age groups [40]. Age is probably an important element to consider in the pathogenesis of these injuries, although the underlying pathophysiology remains unclear. Weight, reduced trunk control and change of movement techniques with growth [41, 42] may be factors related to greater iliopsoas load during kicking, sprinting and change of direction activities in the young.

Several studies have demonstrated the effectiveness of prevention programs in reducing injury incidence, including muscular ones [43, 44]. These non-specific strategies are usually applied to the entire team. Knowledge of the epidemiological profile, particularly at high-performance levels, may allow for the implementation of specific programs tailored to muscle groups [45, 46] with potential beneficial effects for individual players and the team [47].

The significantly longer return-to-football timing among injuries involving the MT/MA supports the hypothesis of a negative prognostic impact of connective tissue involvement in muscle injuries [23, 48, 49]. Therefore, this is one of the factors to consider when diagnosing muscle injuries and formulating a prognosis, especially in hamstring injuries [50]. Data reported in the current study supplement those present in the literature, supporting the application of classification methods for muscle injuries that take into consideration the involvement of connective tissue [48, 51]. Although the involvement of the central aponeurosis of the rectus femoris and the myoaponeurotic junction of the medial gastrocnemius are recognized prognostic factors [31, 52, 53], MT/MA injuries of the quadriceps and calf did not demonstrate a significantly higher impact in terms of days lost in the present study. This is likely due to the reduced absolute number of reported MT/MA injuries in those sites and will require confirmation in further investigations.

The variability in monthly incidence of muscle injuries appears to be primarily attributable to the actual number of muscle injuries, as the overall exposure remained fairly similar in all months of the season. An exception is the month of November, which recorded the lowest number of activity hours. This finding is related to the suspension of the Championship between mid-November 2022 and early January 2023 due to the FIFA World Cup. Clubs suspended training in mid-November, resuming in December. The high incidence of muscle injuries in December, when exposures were mainly due to training sessions and friendly matches, is therefore even more significant. Finally, the peak in incidence towards the end of the season may be attributed to increased intensity and risk-taking behavior as the playoffs and relegation playoffs approached.

The main focus of the current investigation was placed on collecting and analyzing clinically impactful data, such as the number of days lost and the injury burden. The adopted injury definition was designed to maintain a high level of informativeness while minimizing the risk of reporting bias and of disparities caused by the coaching staff management. One-day time-loss issues, which have limited impact and are often managed differently by different Clubs based on the preferences of the coaching staff, were excluded. To our knowledge, this study is the first to analyze first-hand data from a homogeneous population in terms of age and level of sports involvement in elite youth football. Other strengths of the study include the number of teams involved relative to the total participating in this category, the standardized form, the periodic data collection, and the uniformity of shared injury classification criteria inspired by the IOC Consensus.

However, there are several limitations to this study worth highlighting. First, the “time-loss” definition adopted in the present study was slightly different from previously employed definitions. This may imply difficulties in direct comparison with other studies, especially for the absolute numbers (and derived parameters) which may be underestimated. Furthermore, the Under-19 category is selected by age, resulting in significant seasonal population variability. Further studies will be required to determine whether these data are population-specific, age-specific or even coaching staff-specific. Information about the diagnostic methodology used for each injury was not collected and should be considered for future studies. The interruption of the championship due to the FIFA World Cup warrants comparisons regarding monthly muscle injuries incidence with past and future studies. The study cohort was also entirely located in Italy: therefore, generalizations to football players of other countries (i.e., ethnic groups) require further studies and should be made with caution. Finally, injuries and exposures reported during National team commitments were not considered. Hence, for some players, the reported volumes of football activity are slightly lower than what actually occurred.

## Conclusions

For the first time, the characteristics of non-contact muscle injuries in the Italian elite Under-19 football Championship were comprehensively detailed. Hamstrings injury incidence and burden, as well as the proportion of reinjuries, were found to be the highest among muscle sites. Quadriceps accounted as the second most frequent and burdensome muscle injury site. Incidence and burden of adductors injuries were found to be comparable to calf injuries. Iliopsoas injuries represented a noteworthy portion of the total, ranking as the fifth most common site of muscle injury. These findings could potentially be useful for tailoring targeted prevention programs for Under-19 players in conjunction with other risk factors.

Reinjuries and MT/MA injuries represented the potentially most impactful muscle injuries on football availability of young players. Further multi-season studies will be required to assess the degree of population specificity of the current results.

### Electronic Supplementary Material

Below is the link to the electronic supplementary material.

Supplementary Material 1

## Data Availability

The datasets used and/or analyzed during the current study are available from the corresponding Author on reasonable request.

## Abbreviations

SD: Standard Deviation
95% CI: 95% Confidence Interval
MRI: Magnetic Resonance Imaging
MT/MA: Myotendinous/Myoaponeurotic involvement
IOC: International Olympic Committee
RP: Referring Physician
