# Supplementary material for: Epidemiology of Non-Contact Muscle Injuries in the Italian Male Elite Under-19 Football (Soccer) Championship
Source: Sports Med Open. 2024 Jun 21;10:75. doi: 10.1186/s40798-024-00738-0 (PMC11190129; doi:10.1186/s40798-024-00738-0)
Supplement: Supplementary file 1 — Supplementary Material 1 [file 40798_2024_738_MOESM1_ESM.pdf]

To be submitted to Sports Medicine - Open *25th April 2024*

Epidemiology of non-contact muscle injuries in the Italian male elite Under-19 football (soccer) Championship

Massimo Magistrali [1], Luca Stefanini [1,2], Michele Abate [3], Giulio Biancalana [4], Andrea Stegagno [4], Paolo Cugia [5], Piero Candoli [6], Giuseppe Anania [7], Pier Luigi Lucchese [8], Diego Gaddi [10,11,12], Piero Volpi [9,10], Francesco Mariani [2], Lorenzo Boldrini [13], Nicola Filippi [14], Annunziata Cerrone [15], Cristiano Sirtori [16], Paolo Battaglino [17], Guido Bravin [18,19], Emilio Del Fabro [19], Mattia Berti [20,21,22], Eugenio Vecchini [20,21,22], Marco A. Minetto [23]

[1] J|medical, Torino, Italy

[2] Juventus FC, Torino, Italy

[3] IRCCS San Raffaele Hospital, Milano, Italy

[4] FC Bologna 1909, Bologna, Italy

[5] Cagliari Calcio, Cagliari, Italy

[6] FC Cesena, Cesena, Italy

[7] FC Empoli, Empoli, Italy

[8] Frosinone Calcio, Frosinone, Italy

[9] Istituto Clinico Humanitas IRCCS Rozzano, Milano, Italy

[10] FC Internazionale Milano SpA, Milano, Italy

[11] Department of Orthopedics and Traumatology, Policlinico San Pietro, Ponte San Pietro, Bergamo, Italy

[12] Transalpine Center of Pediatric Sports Medicine and Surgery, University of Milano-Bicocca, Monza, Monza-Brianza, Italy

[13] MilanLab Research Department, AC Milan, Milano, Italy

[14]AC Milan, Milano, Italy

[15]SSC Napoli, Napoli, Italy

[16]UC Sampdoria, Genova, Italy

[17]FC Torino, Torino, Italy

[18]SOC Ortopedia e Traumatologia ASUFC, Udine, Italy

[19]Udinese Calcio, Udine, Italy

[20]Clinica Ortopedica, University of Verona, Verona, Italy

[21]Hellas Verona Calcio, Verona, Italy

[22]Department of Knee Surgery, Policlinico Abano Terme, Abano Terme, Italy

[23] Division of Physical Medicine and Rehabilitation, Department of Surgical Sciences, University of Turin, Torino, Italy

Corresponding author

Massimo Magistrali

E-mail: massimo.magistrali@gmail.com

Appendix-1

Definitions

Injury: any physical issue reported by the player resulting from training or a football match, compromising his participation in team activities at least in the **following two days**.

- **Inclusion criteria**:
    - In training:
        - Injury with >2 days lost (day 0 and day 1);
        - Injury leading to non-participation in a match, regardless of the return to football date.
    - In a match:
        - Injury leading to substitution/missed or reduced participation in the match, regardless of the return date.
- **Exclusion criteria**:
    - Injury not related to football sports activity;
    - Training injury with a prognosis of fewer than 2 days (only Day 1 absence) that does not affect participation in matches;
    - Illness/infection;
    - Recurrence of injury during the rehabilitation process, with return to football not yet occurred.

Example: player interrupts training due to **injury** on **Monday** (day 0).

- If the player returns to football on **Wednesday** (day 2) missing one training session, the injury is **not reported**.
- If the player returns to football on **Wednesday** (day 2) missing a **match** played on Tuesday, the injury is **reported**.
- If the player returns to football on **Thursday** (day 3) missing two training sessions, the injury is **reported**.

Match: **official competition** and **friendly matches between two different Clubs** are considered. Friendlies within the same club are considered as part of the training exposure. Also, please note if the timing differs from 90 minutes (e.g., a friendly match with two halves of 40 minutes).

Return to football date (RTF): the date of the first unrestricted training session with the team or a match.

Some clarifications:

- Multiple injuries to the same player in the same event should be reported as a single injury with multiple diagnoses.
- Prompt completion after each injury is recommended to avoid *recall bias*.

Recurrence of injury: an injury of the same type and at the same site as a previous one that occurs after the return to unrestricted sports activity. They are classified as:

- Early: within 2 months after RTF;
- Late: 2-12 months after RTF;
- Delayed: more than 12 months after RTF.
